# Deep Survival Analysis With Clinical Variables for COVID-19

**DOI:** 10.1109/JTEHM.2023.3256966

**Published:** 2023-03-14

**Authors:** Ahmad Chaddad, Lama Hassan, Yousef Katib, Ahmed Bouridane

**Affiliations:** School of Artificial IntelligenceGuilin University of Electronic Technology 71207 Guilin Guanxgi 541004 China; 2Laboratory for Imagery, Vision, and Artificial IntelligenceEcole de technologie Superieure 14849 Montreal QC H3C 1K3 Canada; College of MedicineTaibah University 123305 Madinah 42353 Saudi Arabia; Centre for Data Analytics and CybersecurityUniversity of Sharjah 59105 Sharjah United Arab Emirates

**Keywords:** COVID-19, CNN, clinical variables

## Abstract

Objective: Millions of people have been affected by coronavirus disease 2019 (COVID-19), which has caused millions of deaths around the world. Artificial intelligence (AI) plays an increasing role in all areas of patient care, including prognostics. This paper proposes a novel predictive model based on one dimensional convolutional neural networks (1D CNN) to use clinical variables in predicting the survival outcome of COVID-19 patients. Methods and procedures: We have considered two scenarios for survival analysis, 1) uni-variate analysis using the Log-rank test and Kaplan-Meier estimator and 2) combining all clinical variables ( $n$=44) for predicting the short-term from long-term survival. We considered the random forest (RF) model as a baseline model, comparing to our proposed 1D CNN in predicting survival groups. Results: Our experiments using the univariate analysis show that nine clinical variables are significantly associated with the survival outcome with corrected p < 0.05. Our approach of 1D CNN shows a significant improvement in performance metrics compared to the RF and the state-of-the-art techniques (i.e., 1D CNN) in predicting the survival group of patients with COVID-19. Conclusion: Our model has been tested using clinical variables, where the performance is found promising. The 1D CNN model could be a useful tool for detecting the risk of mortality and developing treatment plans in a timely manner. Clinical impact: The findings indicate that using both Heparin and Exnox for treatment is typically the most useful factor in predicting a patient’s chances of survival from COVID-19. Moreover, our predictive model shows that the combination of AI and clinical data can be applied to point-of-care services through fast-learning healthcare systems.

## I. Introduction

The COVID-19 pandemic, which began in 2019, has rapidly spread across the globe and was officially declared a pandemic by the World Health Organization on March 11, 2020 [1]. This infectious disease is characterized by symptoms such as fever, fatigue, dry cough, and respiratory problems, and as of September 24, 2021, it has resulted in over 230 million confirmed cases and more than 4.73 million deaths worldwide. The United States has been particularly affected, with over 43.53 million cumulative cases [2]. Despite recent research efforts to identify factors that contribute to COVID-19-related mortality, there are few studies investigating the relationship between clinical procedures, such as tests and treatments, and patient survival outcomes.

Previous studies have primarily focused on computer-aided diagnosis methods for detecting COVID-19 [3], [4], [5] (and/or tracking its spread [6]), which include temperature readings [7], molecular analysis such as RT-PCR (reverse transcription polymerase chain reaction: RT-PCR), chest CT scans, and chest radiographs [8], [9]. Some researchers have also used 1D CNN to diagnose respiratory diseases associated with COVID-19 using human respiratory sounds such as voice, cough, and breath [10], [11].

In [12], COVID-19-related variables such as confirmed cases, recoveries, and deaths were used with 1D CNN to forecast COVID-19 trends in France. Meanwhile, in [13], a combination of statistical features and 1D CNN was developed to forecast COVID-19 time series. However, there are still several clinical variables related to COVID-19 patients and their survival that remain unexplored. Exploring these variables through automated means could help improve clinical procedures in patient treatment and enhance survival outcomes. In this regard, artificial intelligence (AI) models can have a significant impact on predicting clinical outcomes, such as survival. Specifically, *our objective is to build a prognostic model for COVID-19 patients using available clinical variables.*

AI has played an increasingly important role in healthcare, particularly in the context of big data analysis [14], [15], [16]. In the fight against COVID-19, AI has been used for a variety of purposes, such as predicting infection [17] and identifying poor outcomes in patients with the disease [18]. Machine learning approaches have also been used to predict hospital mortality and identify key predictors, including clinical characteristics such as oxygen saturation readings, demographics, and patient comorbidity information [19], [20], [21], [22]. However, one challenge is the identification of significant markers among the numerous clinical variables associated with COVID-19. Therefore, the aim of this study is to analyze the relationships between clinical variables and the survival outcome of patients with COVID-19. A predictive model can be beneficial in this context by providing clinicians with accurate treatment suggestions and enabling the analysis of large quantities of data in a timely and accurate manner, leading to more effective treatments.

The main contributions of our work are as follows:

• Our study conducts both univariate and multivariate analyses to examine the association between clinical variables and survival outcome of COVID-19 patients.
• Our study highlights the significance of clinical variables as predictors, both predictive and non-predictive, in forecasting survival outcomes. This was demonstrated by using the RF model as a baseline. model.
• Our work also introduces a novel 1D CNN model that outperforms state-of-the-art techniques in predicting the survival groups of COVID-19 patients, resulting in improved performance metrics.

Based on our experimental results, we were able to achieve significant classification performances in distinguishing between short-term and long-term survival of patients with COVID-19. Our study provides a comprehensive analysis of the relationships between clinical variables, treatment options, and the survival outcome of COVID-19.

The structure of the paper is organized as follows: Section II presents related works on the clinical variables and survival of patients with COVID-19. Section III describes the clinical variables with 1D CNN and RF models for the classification tasks. The experimental results are presented in Section IV, and the main findings are discussed in Section V. Finally, Section VI summarizes the key contributions and results of our work, highlighting the significance of our proposed model for predicting survival outcome in COVID-19 patients.

## II. Related Work

Previous studies have shown that elderly patients tend to have poor outcomes [23]. For example, patients infected with COVID-19 were studied and a significant difference in age was found between the survival groups. Using the multivariate analysis, age was a predictive variable that relates to prognosis in patients with COVID-19. In [24], heparin treatment was able to extend survival of COVID-19 patients. This finding is proved by the safety and effectiveness of heparin for COVID-19 in [25]. Similarly, treatment with enoxaparin was able to extend survival [26]. Convalescent plasma therapy is also considered, and patients with COVID-19 are associated with improved outcomes [27]. Another study shows that older people and children have higher infection rates [28], and the risk of death increases independently with age [29]. In addition, hormones such as N-terminal hormone (NT) -pro BNP (NT-proBNP) probably help doctors screen high-risk patients with COVID-19 [30]. In [31], the troponin value increases more significantly in patients with severe infection than in those with mild symptoms. In [32], it was found that acute kidney injury is associated with a poor prognosis in patients with COVID-19. In [33], in addition to age, three biochemical parameters were related to mortality regardless of other comorbidities. However, *no study yet consists of a comprehensive analysis using public clinical variables of COVID-19 for further survival exploration.*

From a technical point of view, many algorithms are widely applied to predict the COVID-19 [34]. However, the survival outcome of COVID-19 patients using CNNs is still limited. For example, a machine learning algorithm is used to predict the mortality risk of COVID-19 [17]. In [35], many algorithms with statistical analysis approaches used for discharge-time prediction of COVID-19 cases. Their findings show that the Gradient Boosting survival model outperforms other models of patient survival. Additionally, cox regression with auto-encoder are used to study the survival outcome for COVID-19 and predict the most important symptoms (features) affecting survival probability [36]. A new study, based on deep learning technology, which incorporates multiple, time-fixed data, longitudinal non-image data and longitudinal images, shows the capacity to predict the survival outcome of COVID-19 [37]. Compared against previous contributions, our approach consists of two main scenarios: 1) apply the univariate analysis to measure the relationships between clinical variables and survival outcome, and 2) propose 1D CNN to improve performance metrics compared to the random forest model in predicting the survival groups of COVID-19. To our knowledge, no comprehensive study has yet investigated the relationships between clinical variables, treatment options, and their relationships with the survival outcome of COVID-19 patients.

## III. Materials and Methods

**FIGURE 1.**
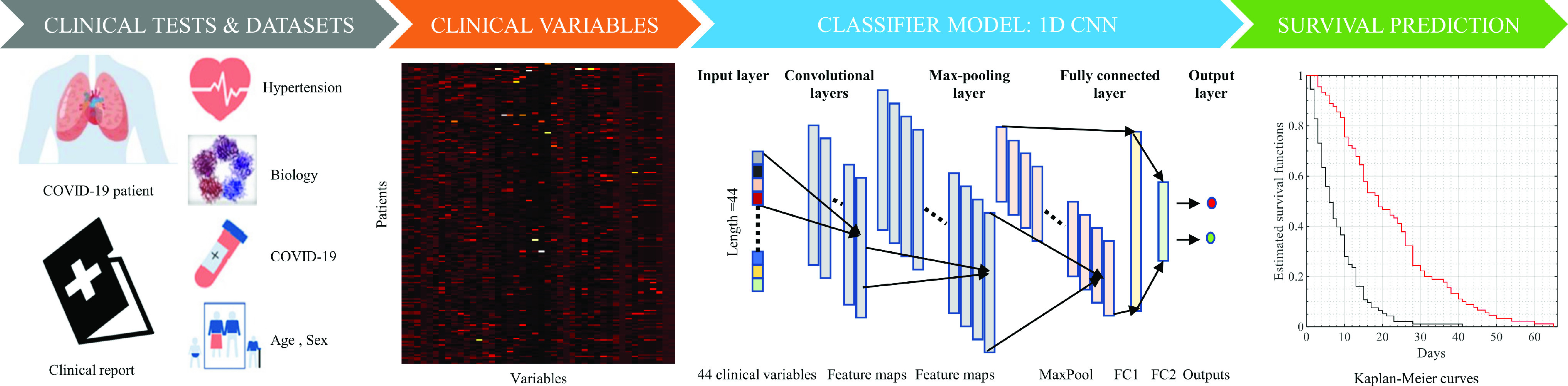
Our study presents a survival analysis-based pipeline for predicting the survival outcome of COVID-19 patients using a set of 44 clinical variables. The pipeline involves (1) aggregating clinical data sets from multiple tests and treatments, (2) selecting relevant clinical variables, and (3) using a proposed architecture that includes two convolutional layers, a max-pooling layer, two fully connected layers (FC1 and FC2), and an output layer. The pipeline is evaluated using the log-rank test and Kaplan-Meier estimator to compare survival between two groups of COVID-19 patients.

The survival analysis pipeline is illustrated in Figure 1. In this study, we collected data on 44 clinical variables along with the survival outcome of COVID-19 patients. We performed statistical and classification analyses to investigate the relationship between these variables and survival outcomes using two approaches, namely univariate analysis (Log-rank test and Kaplan-Meier estimator) and multivariate analysis (1D CNN and random forests). The subsections below provide further details on these analyses.

### A. Datasets and Clinical Variables

We collected clinical data from 1384 COVID-19 patients through The Cancer Imaging Archive (TCIA) [38]. The patients included in these data had tested positive for COVID-19 and did not have cancer. The patients had already been de-identified by TCGA/TCIA, and our study did not require any institutional review board or Health Insurance Portability and Accountability Act approvals. Stony Brook University had obtained these datasets from COVID-19 patients.^1^ These datasets contained clinical data for each patient, such as their diagnosis, procedures, laboratory test results, COVID-19-specific data values, and a range of derived data elements that were analyzed in our study. TCIA [38] provided a detailed list of clinical variables and TCGA IDs for each patient. For survival analysis, we used both uncensored patients ( $n$=183; from the date of their first COVID-19 diagnosis until their date of death) and censored patients ( $n$=1201; from the date of their first COVID-19 diagnosis until their discharge from the hospital), along with their corresponding clinical features. We collected 44 prominent clinical variables (i.e., $v1$, $v2$,..., $v44$) and their corresponding survival outcomes. These variables are: $v1:$ age, $v2:$ gender, $v3:$ acute hepatic injury during hospitalization, $v4:$ acute kidney injury during hospitalization, $v5:$ urine protein, $v6:$ smoking, $v7:$ oral temperature, $v8:$ oxygen saturation in arterial blood by pulse oximetry, $v9:$ respiratory rate, $v10:$ heart rate beat to beat by EKG, $v11:$ Systolic blood pressure, $v12:$ mean blood pressure by noninvasive, $v13:$ leukocytes corrected for nucleated erythrocytes in blood by automated count, $v14:$ neutrophils in blood by automated count, $v15:$ lymphocytes in blood by automated count, $v16:$ sodium in serum or plasma, $v17:$ aspartate aminotransferase in serum or plasma, $v18:$ alanine aminotransferase in serum or plasma by no addition of P-5’-P, $v19:$ creatine kinase in serum or plasma, $v20:$ lactate in serum or plasma, $v21:$ troponin T.cardiac in serum or plasma, $v22:$ natriuretic peptide.B prohormone N-Terminal in serum or plasma, $v23:$ procalcitonin in serum or plasma by immunoassay, $v24:$ fibrin D-dimer DDU in platelet poor plasma by immunoassay, $v25:$ ferritin in serum or plasma, $v26$ C reactive protein in serum or plasma, $v27:$ hemoglobin A1c/hemoglobin total in blood, $v28:$ body mass index (BMI), $v29:$ sodium in serum or plasma, $v30:$ potassium in serum or plasma, $v31:$ chloride in serum or plasma, $v32:$ bicarbonate in serum or plasma, $v33:$ urea nitrogen in serum or plasma, $v34:$ creatinine in serum or plasma, $v35:$ glomerular filtration rate/1.73 sq M.predicted, in Serum, plasma or blood by creatinine-based formula (CKD-EPI), $v36:$ pH of arterial blood adjusted to patient’s actual temperature, $v37:$ erythrocyte sedimentation rate, $v38:$ glucose in serum or plasma, $v39:$ cholesterol in LDL in serum or plasma by calculation, $v40:$ cholesterol in VLDL in serum or plasma by calculation, $v41:$ triglyceride in serum or plasma, $v42:$ cholesterol in HDL in serum or plasma, $v43:$ therapeutic exnox boolean, and $v44:$ therapeutic heparin boolean.^1^https://doi.org/10.7937/TCIA.BBAG-2923

To handle missing variable values, we employed a decision tree-based imputation method. This involves constructing a decision tree model for each variable that has missing values, using the remaining variables in the dataset as predictors. The model is trained on a subset of the data that contains no missing values, and then used to estimate the missing values for each variable. These estimated values are then used to substitute the missing values. Our choice of the decision tree method, as mentioned in [39], was due to its ability to handle both numerical and categorical variables with minimal bias, making it a preferable option over other machine learning-based imputation techniques.

### B. Statistical and Classification

#### 1) Survival Analysis:

In univariate analysis, we partitioned the patients into two survival groups (below-median or above-median clinical variables). We then performed a log-rank test and Kaplan-Meier estimator to compare between the two survival groups. In multivariate analysis, we applied the log-rank test and Kaplan-Meier estimator to compare between predicted groups (lower-than-median and higher-than-median survival outcome) for patients with COVID-19 [40]. To account for multiple comparisons of clinical variables, all p-values obtained from significance tests were corrected simultaneously according to the Holm-Bonferroni procedure [41]. We considered the variables statistically significant when corrected p < 0.05.

#### 2) Classification:

We divided the COVID-19 patients into two groups based on their survival time: below median-survival (short survival) and above median-survival (long survival). We used all 44 clinical variables as input for a classifier model, which included a 1D CNN and/or a random forest. Initially, we used the random forest classifier with 500 decision trees and 10 maximum tree depths as a baseline model. We chose this model due to limited samples and its ability to assess the importance of predictive variables [42], [43].

We used two validation scenarios to evaluate the classifier models: 1) leave-one-out cross-validation (LOOCV), where we divided the training variables into $n$ samples and tested the RF classifier on a single sample at each iteration while using the remaining $n-1$ samples to train the model. We evaluated the cross-validation-based classifier models based on the average iterations $n$ for metrics such as AUC-ROC, $Accuracy$, $Sensitivity$, $Specificity$, and $Precision$. 2) We randomly split the samples into training and test sets to provide an unbiased evaluation of the model fitted on the training samples. The overall performance of the model was measured by calculating the average ± standard deviation obtained over 10 iterations for metrics such as AUC-ROC, $Accuracy$, $Sensitivity$, $Specificity$, and $Precision$.

\begin{align*} \mathrm { \textit {Accuracy}} &= \frac {TP + TN}{N} \times 100 \tag{1}\\ \mathrm { \textit {Sensitivity}} &= \frac {TP}{TP + FN} \times 100 \tag{2}\\ \mathrm { \textit {Specificity}} &= \frac {TN}{TN + FP} \times 100 \tag{3}\\ \mathrm { \textit {Precision}} &= \frac {TP}{TP+FP} \times 100\tag{4}\end{align*}

where $TP$ ( $TN$, resp.) is the number of correctly predicted short-term survival (long-term survival) examples, $FP$ ( $FN$, resp.) the number of examples incorrectly predicted as short-term survival (long-term survival, resp.), and $N$ the total number of examples. Using these metrics, we can measure the AUC-ROC and plot *True positive rate* (*Sensitivity*) versus *False positive rate* (1- *Specificity*) at different decision thresholds.

### C. Proposed 1D CNN Classifier

The objective of the proposed end-to-end 1D CNN architecture is to automatically learn essential clinical features from clinical variables and predict the survival groups of COVID-19 patients. To achieve this, the architecture utilizes trainable convolutional layers to learn an appropriate representation of the clinical variables. Each neuron in a layer is only connected to a small region of the previous layer, which is referred to as the “region of connectivity.” For instance, the input to the 1D CNN is an array that represents clinical variables denoted as $X$. The network is designed to learn a set of parameters $\Theta $ that maps the input to the prediction $T$. The hierarchical feature extraction is performed through this mapping process.

\begin{equation*} \mathrm {T} = f_{l}(X_{l}|\Theta _{l}) =h(W\bigotimes X_{l} + b), \Theta _{l}=[W,b]\tag{5}\end{equation*}

where $\bigotimes $ denotes the convolution operation, The input matrix $X_{l}$ consists of $N$ feature maps and is in a two-dimensional format. The set of one-dimensional kernels, denoted by $W$, consists of $N$ elements and is utilized to extract a new set of features from the input array. The bias vector is represented by $b$, and the activation function, such as ReLU (Rectified Linear Unit), is denoted by $h$. After the final convolutional layer, its output is flattened and subsequently employed as input for multiple stacked fully connected layers. This process can be characterized as follows:

\begin{equation*} \mathrm {T} = f_{l}(X_{l}|\Theta _{l}) =h(WX_{l} + b), \Theta _{l}=[W,b]\tag{6}\end{equation*}

The output layer follows the fully connected layer and is responsible for making predictions. In this model, we propose a 1D CNN architecture that takes 44 clinical variables as input, with an image size of $1\times 44$. The architecture of the proposed 1D CNN is as follows.

• Input: 1D image size = $1\times 44$,
• Convolution layer 1 (Filters = 64, size = $1\times 3$; Activation = ReLU),
• Convolution layer 2 (Filters = 128, size = $1\times 3$; Activation = ReLU, Average pooling = $1\times 2$),
• Fully connected layer 1 (Activation = ReLU; Output = (1) $\times 128$),
• Fully connected layer 2 (Activation = ReLU; Output = (1) $\times 64$),
• Output layer (Activation = Softmax; Output = $1\times 2$).

The 1D CNN architecture comprises six layers and uses a training/validation/testing dataset split of 110/12/61, with a learning rate of 0.0005, a cross-entropy loss function, and stochastic gradient descent optimization with a momentum value of 0.9. The goal of applying the 1D CNN was to classify samples into short-term and long-term survival groups. To compare the predicted survival outcomes between the groups, we used the Log-rank test and Kaplan-Meier estimator. Additionally, we compared the predicted survival groups obtained from different models, such as the 1D CNN versus the RF model as a baseline. To determine the significance value, we calculated the chi-square test as described in [44]. All processing and analysis steps were carried out using Matlab’s Deep Learning, Statistics, and Machine Learning Toolbox.

## IV. Result

### A. Variables Associated with Survival

We evaluated the association between individual clinical variables and the survival of COVID-19 patients by conducting a log-rank test and Kaplan-Meier estimator on a sample of deceased COVID-19 patients (total sample size, $n$=183). We find that nine clinical variables (i.e., *age; acute kidney injury during hospitalization; troponin T.cardiac in serum or plasma natriuretic peptide.B prohormone; N-Terminal in serum or plasma; urea nitrogen in serum or plasma; creatinine in serum or plasma; glomerular filtration rate/1.73 sq M.predicted (Volume rate/area) in serum, plasma or blood by creatinine-based* formula (CKD-EPI); therapeutic exnox boolean; therapeutic heparin boolean) are significantly associated with survival outcome with corrected p < 0.05. We observe that the most significant variable is related to *Therapeutic heparin Boolean (v44)*. Specifically, a longer survival was associated with a higher variable value for $v44$ (i.e., median survival: 7 versus 19 days, Hazard Ratio (HR) of 3.59, Confidential Intervals (CI) of 2.55-5.04 and corrected p of $1.36\times 10^{-8}$. Table 1 reports the significant clinical variables with corrected p < 0.05. The rest of the clinical variables were not significant, with corrected p $>$ 0.05.

**TABLE 1:** Comparison of COVID-19 Patient Survival Grouped by Individual Variable Values: Summary of Clinically Significant Variables (Corrected p-Value < 0.01)

Variables ( $v$)	$n$	Median survival		HR	CI	p-value	corrected p-value
		$\boldsymbol {\le }$	$\boldsymbol {>}$				
$v1$	183	15	9	0.43	0.31–0.61	$1.59\times \,\,10^{-6}$	$6.34\times \,\,10^{-5}$
$v4$	yes=126, no=57	7	15	2.92	1.95–4.39	$3.63\times \,\,10^{-7}$	$1.52\times \,\,10^{-5}$
$v21$	166	15	7	0.42	0.3–0.6	$1.5\times \,\,10^{-6}$	$6.13\times \,\,10^{-5}$
$v22$	144	15	7	0.43	0.29–0.62	$1.26\times \,\,10^{-5}$	0.0005
$v33$	182	15	9	0.54	0.39–0.74	0.0002	0.007
$v34$	182	15	8.5	0.50	0.36–0.69	$4.17\times \,\,10^{-5}$	0.0016
$v35$	178	9	15	1.98	1.43–2.74	$5.27\times \,\,10^{-5}$	0.002
$v43$	81	7	19	2.91	2.1–4.05	$3.25\times \,\,10^{-10}$	$1.4\times \,\,10^{-8}$
$v44$	79	7	19	3.59	2.55–5.04	$3.08\times \,\,10^{-10}$	$1.36\times \,\,10^{-8}$

### B. Clinical Signature Related to Survival

**FIGURE 2.**
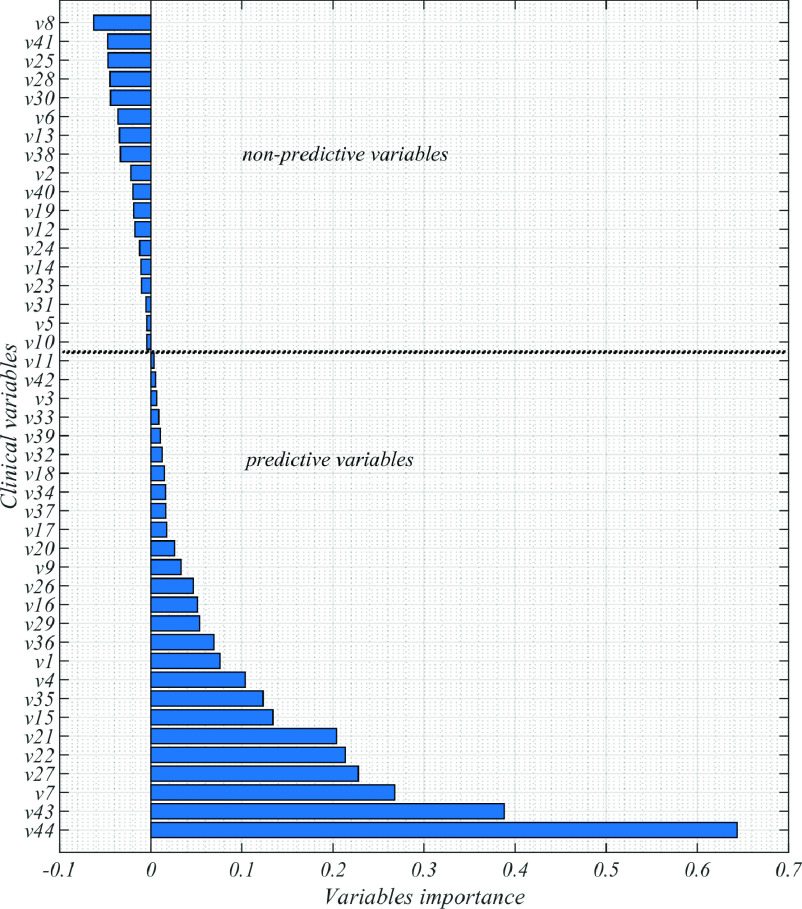
Bars of individual variables’ importance for predicting the survival group of censored COVID-19 patients ( $n$ = 183). Importance values represent the average increase in prediction error obtained by permuting the values of individual variables across out-of-bag observations.

We considered the RF model inside LOOCV to classify the censored patients ( $n$ = 183), in the shorter-term or longer-term survival groups. Therefore, the classifier model using all clinical variables (*v1, v2,...,v44*) shows a significant difference between these two groups, with a corrected p < 0.05. Specifically, the RF model shows an AUC, Accuracy, Sensitivity, Specificity, Precision, HR, CI, and p value of 77.64%, 73.77%, 73.68%, 72.22%, 4.73, 3.28–6.82 and p = $2.2\times 10^{-16}$, respectively. Using the RF model, we measured the importance of variables. Figure 2 shows that 26 clinical variables have positive predictive values (i.e., importance value $>$ 0) while 18 variables have negative values. Once again, the importance of variables in the RF model demonstrates that the *Therapeutic heparin Boolean* is the highest predictive variable.

### C. Deep Survival Analysis Based Clinical Variables

**FIGURE 3.**
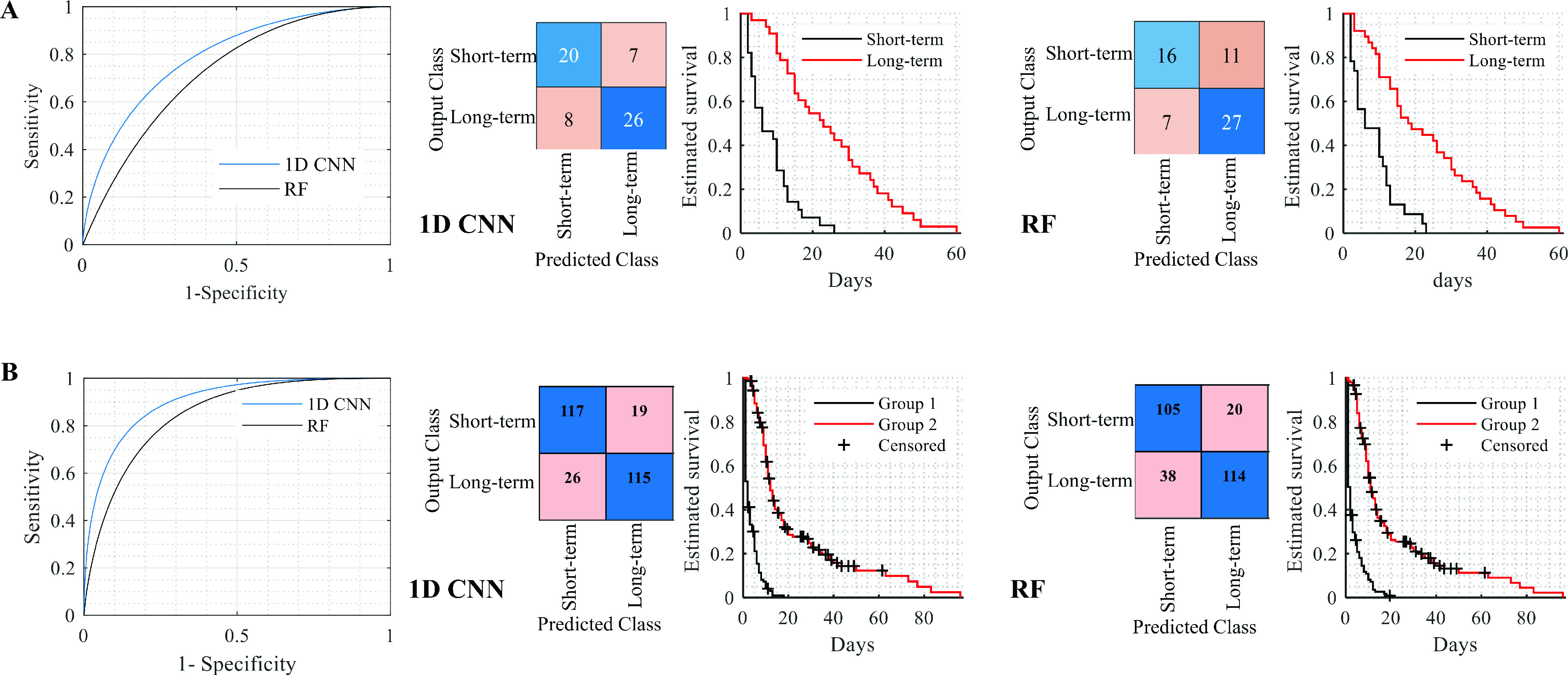
Performance metrics derived from 1D CNN and RF models for predicting short-term and long-term survival groups (A: censored patients ( $n$ = 183), training/testing = 122/61; B: Training/testing = 1107/277). ROC (left), confusion matrix, log-rank and Kaplan-Meier measure the significance of the predicted survival groups from classifier models.

Our deep CNN model was trained using clinical variables, with 110 training samples and 12 validation samples. We then tested the model using 61 samples and found that its performance metrics were significantly better than those of the random forest model in classifying short-term and long-term survival groups of COVID-19 patients. Specifically, the 1D CNN model had an AUC-ROC of 79.24%, accuracy of 75.40%, sensitivity of 76.47%, specificity of 74.07%, precision of 78.78%, HR of 7.48, HRci of 3.70–15.10, and $p$-value of $5.3\times 10^{-8}$, while the random forest model had an AUC-ROC of 72.48%, accuracy of 70.49%, sensitivity of 79.41%, specificity of 59.25%, precision of 71.05%, HR of 6.19, HRci of 2.95–13.01, and $p$-value of $3.6\times 10^{-6}$. These results are shown in Figure 3A and Table 2, and indicate that the 1D CNN model is a superior classifier of short-term and long-term survival groups in COVID-19 patients, with a $p$-value less than 0.05.

**TABLE 2:** Metrics for Predicting COVID-19 Patient Survival Group: Analysis of Performance for 61 Patients

Classifier	AUC	Accuracy	Sensitivity	Specificity	Precision	HR	HRci	p value
RF	72.48± 3.27	70.49± 0.17	79.41± 4.24	59.25± 4.67	71.05± 3.39	6.19	2.95–13.01	$3.6\times 10^{-6}$
1D CNN	79.24± 3.53	$75.40 \pm 0.15$	76.47± 1.38	74.07± 0.23	78.78± 0.48	7.48	3.70–15.10	$5.3\times 10 ^{-8}$

### D. Impact of Censored Patients

Using the proposed 1D CNN and RF models, we considered the survival analysis for whole COVID-19 patients ( $n$ = 1384: 183 uncensored and 1201 censored). We partitioned the whole samples ( $n$= 1384) into two survival groups (below and above median survival = 6 days) with training/testing of 1107/277 samples. Once again, the 1D CNN performance metrics (AUC-ROC = 90.25%, Accuracy = 83.75%, Sensitivity = 85.82%, Specificity = 81.81%, Precision = 81.56%, HR = 12.70, HRci = 8.85–18.23 and p value = 0) are significantly better than the random forest model (AUC-ROC = 84.78%, Accuracy = 79.06%, Sensitivity = 85.07%, Specificity = 73.42%, Precision = 75.00%, HR = 9.23, HRci = 6.45–13.22 and p value = 0) to predict survival groups with p < 0.05 (i.e., Figure 3B and Table 3). Using the classifier model to predict survival of censored patients may be validated when we applied the log-rank test and the Kaplan-Meier estimator to assess the difference of the predicted groups.

**TABLE 3:** Performance Metrics for Predicting the Survival Group of 277 COVID-19 Patients

Classifier	AUC	Accuracy	Sensitivity	Specificity	Precision	HR	HRci	p value
RF	84.78± 2.19	79.06± 1.90	85.07± 3.82	73.42± 3.97	75.00± 0.93	9.23	6.45–13.22	$< 10^{-10}$
1D CNN	90.25± 1.38	83.75± 3.39	85.82± 3.27	81.81± 0.81	81.56± 0.59	12.70	8.85–18.23	$< 10^{-10}$

**FIGURE 4.**
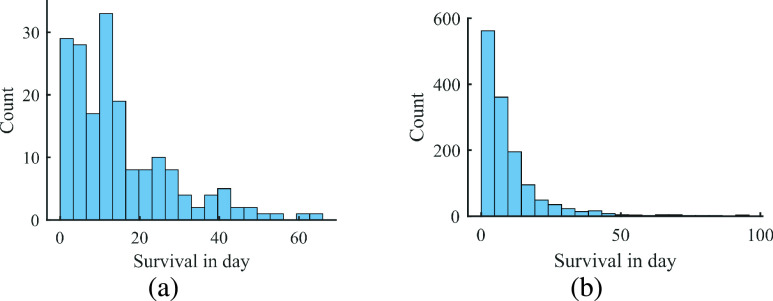
Histogram of survival lengths for patient with COVID-19 (days). (right) Uncensored ( $n$=183) distribution. (left) whole cases ( $n$=1384).

For a censored patient (i.e., if the person was alive at the end of the study or was lost to follow-up at any time during this study), the survival imputation technique was used for the classifier model. For censored patients ( $n$ = 1201), we considered the average survival time of the remaining patients with a time-to-death greater or equal to their own, as of the time at the last visit. This result can be explained by the nature of survival distributions, which can be appreciated in Fig 4

### E. Comparison with Other State-Of-Art Models

**FIGURE 5.**
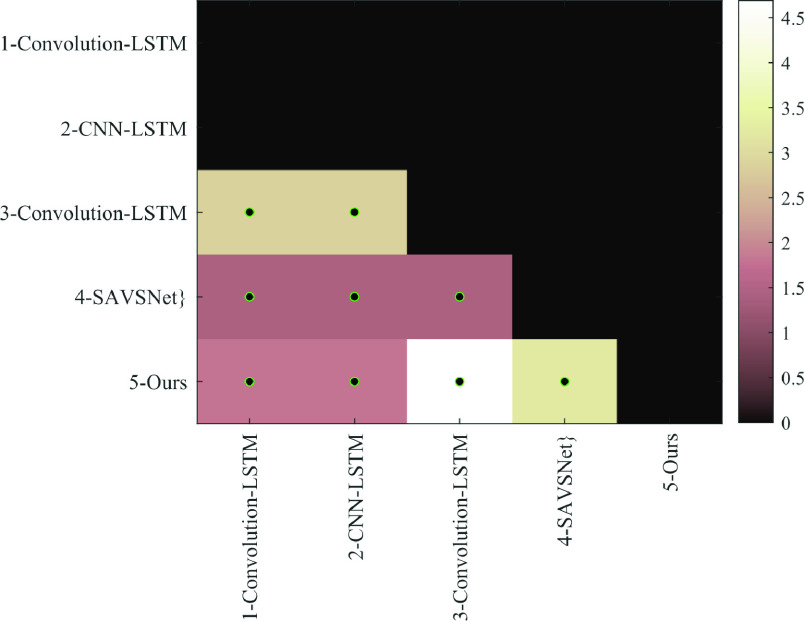
Heatmap of significance value ( $-\log _{10} (\textrm {p-value})$) from chi-square test to measure the differences between two CNN model of the predicted groups (e.g., predicted groups using Convolution-LSTM [45] versus SAVSNet [48]), green-black circle represents the significance difference between two predictive models in predicting the shorter-term from longer-term survival with $\text{p}\,< \,0.05$.

To evaluate the impact of the proposed 1D CNN, we considered also another CNN architectures using whole COVID-19 patients (n = 1384: 183 uncensored and 1201 censored). We randomly partitioned the samples (n= 1384) into two survival groups (below and above median survival = 6 days) with training/testing of 1107/277 samples. In Table 4, we compared our 1D CNN model’s results with those of other 1D CNN architectures for classifying short and long survival groups. Our findings suggest that our model’s performance is comparable to state-of-the-art models, as described in [45], [46], [47], and [48]. Specifically, we achieved a higher AUC (90.25% versus 84.36–88.10%) and accuracy (83.75% versus 79.06–81.94%) than these previous CNN architectures. Our 1D CNN model resulted in an AUC improvement of 3–6% over the other 1D CNN architectures for predicting the survival group of COVID-19 patients. Based on the chi-square test, the improvement of our method over other approaches tested is significant, with p < 0.05 (Fig. 5).

**TABLE 4:** Performance Comparison of 1D CNN Models for Predicting the Survival Group of 277 COVID-19 Patients

Classifier	AUC	Accuracy	Sensitivity	Specificity	Precision	HR	HRci	p value
1-Convolution-LSTM [45]	87.86±5.88	81.94±1.78	79.85±5.70	83.91±1.70	82.30±6.50	8.53	6.11–11.91	$< 10^{-10}$
2-CNN-LSTM [46]	88.10±5.25	81.94±1.78	85.07±3.54	79.02±4.89	79.16±6.23	10.19	7.16–14.50	$< 10^{-10}$
3-Convolution-LSTM [47]	84.36±2.46	79.06±5.81	80.59±4.09	77.62±3.84	77.14±6.42	7.52	5.37–10.54	$< 10^{-10}$
4-SAVSNet [48]	86.48±0.53	80.50±0.37	70.14±3.71	90.20±5.45	87.03±6.53	7.48	5.45–10.27	$< 10^{-10}$
5-Ours	90.25±1.13	83.75±5.56	85.82±2.17	81.81±3.69	81.56±1.15	12.70	8.85–18.23	$< 10^{-10}$

### F. 1D CNN Based Analysis

To analyze the impact of 1D CNN, we use the same conditions in training 1D CNN with training samples (train/test=110/12) of uncensored patients to predict the test samples ( $n$ = 61) of short-term and long-term survival groups. We performed two other 1D CNN with different convolutional layers as follows:

• 6 layers: Input image, 3 Convolutional layers (Filters = 64, 128 and 128) and 2 Fully connected layers.
• 9 layers: Input image, 6 convolution layers (Filters = 32, 64, 128, 32, 64 and 128) and 2 Fully connected layers.

Table 5 reports the performance metrics of 61 test samples with 6 and 9 layers of 1D CNN. We found that the 1D CNN with 6 and 9 layers are significantly predicting the survival groups with p-value of $4.1\times 10 ^{-8}$ and $2.7\times 10 ^{-8}$, respectively. We note that the convolutional layers (filters) extract features that were used to adjust their weights.

**TABLE 5:** Performance Metrics of Two Different 1D CNN Architectures for Predicting the Survival Group of the 61 Test COVID-19 Patients

Classifier	AUC	Accuracy	Sensitivity	Specificity	Precision	HR	HRci	p value
1D CNN with 6 layers	78.97±3.15	75.75±0.58	83.33±1.60	66.66±6.39	75.00±1.06	6.21	4.5–12.34	$4.1\times 10 ^{-8}$
1D CNN with 9 layers	75.22±0.74	72.72±6.73	83.33±0.03	60.00±5.42	71.42±5.72	4.83	4.1–14.42	$2.7\times 10 ^{-8}$

## V. Discussions

With COVID-19, the ability to develop an AI model is critical, through a predictive modeling routinely used is often underdiagnosed, so a deep learning model using clinical variables has remarkable potential. We note that a survival analysis cannot directly combat COVID-19 (i.e., treatment). However, a deep CNN model allows us to monitor the severity of virus infection with related factors, thus allowing clinicians to make the required policies timely. Many predictive models have been used to predict COVID-19 cohorts with varying results; some of them have previously been developed to predict the presence of COVID-19 using chest CT and X-ray scans [34], [49]. Investigation of the association between survival and treatment options or other molecular analysis is still limited.

In this work, we propose a predictive model based on 1D CNN using 44 clinical variables as input to predict the survival outcome of patients with COVID-19. Our findings demonstrated that two clinical variables, heparin and exnox are the most significant indicators of survival prediction. This is consistent with previous literature on heparin and enoxaparin that were associated with long survival in COVID-19 patients [24], [25], [26]. Specifically, this study investigated all prominent clinical variables related to COVID-19. The other studies almost focused on an early warning model to predict COVID-19 in hospital mortality [50], [51]. Experiments with many sizes of filters (Table 4 and Table 5) showed significant differences between predicted survival groups with p < 0.05. Comparing to other 1D CNN architectures, the proposed 1D CNN shows superior performance. However, the proposed 1D-CNN does not improve the performance metrics when more convolutional layers (filters) are implemented in the same model. This means that the model was not learning efficiently from the collected features as the number of filters increased from the optimal value. It then provides no improvement for testing sample prediction performance, as reported in Table 5 comparing to Table 2.

The advantage of using clinical variables as input into the deep CNN model is that all of these variables can be synergized to improve the performance in survival prediction. The novelty of our study is represented by showing the impact of each of clinical variables in predicting the survival outcome and offered a simple 1D CNN implementation for clinical tasks (i.e., survival, etc.). Another major advantage is related to compact configuration of 1D CNN that perform only 1D convolutions. It shows good performances on a limited amount of data sets ( $n=1384$), low computational requirements (compared to 2D CNN and 3D CNN architectures), and a good ability to extract relevant features from clinical variables. More details about 1D CNN benefit is discussed in [52]. In the case of exploiting further CNN architectures, there is still ample room to improve the performance metrics by involving the corresponding CT-Scan and X-ray images.

In this work, we used the median survival time because it provides a balance of classes. However, other strategies for this task could be considered, such as dividing patients into a potentially larger number of clinically relevant survival periods caused by different causes of COVID-19. Another option is to formulate the problem as a regression task rather than a classification process and predict the risk of death of patients [53] or rank the survival time of different patients [54]. In addition, this study only focuses on COVID-19 without to consider the new variants and/or sub-variants like Omicron and Delta [55]. More investigation in this direction will improve the accuracy of survival analysis. More importantly, efforts must be directed to automatically exploit lung image segmentation with public access to these labels from the TCIA website. As such, this may simplify our understanding of COVID-19 when clinicians estimate the survival group from chest CT and / or X-ray images and provide accurate treatment to achieve the goal of personalized medicine.

## VI. Conclusion

This study presents a 1D CNN model to predict the survival outcomes of patients with COVID-19 while investigating the impact of clinical variables on the prediction. The findings revealed that heparin treatment significantly influenced the survival outcome. Despite the promising results demonstrated by the 1D CNN model in survival prediction, the performance may be improved by integrating new clinical variables and imaging features. Moreover, we plan to present a part of the results at [56]. Furthermore, the study proposes the development of a novel model, the CNN+RNN, which can model temporal sequences with images. This model is expected to offer more options of data types and to achieve higher performance metrics than the current model.
